# Effects of Low-Dose versus High-Dose **γ**-Tocotrienol on the Bone Cells Exposed to the Hydrogen Peroxide-Induced Oxidative Stress and Apoptosis

**DOI:** 10.1155/2012/680834

**Published:** 2012-08-21

**Authors:** Nizar Abd Manan, Norazlina Mohamed, Ahmad Nazrun Shuid

**Affiliations:** ^1^Department of Pharmacology, Faculty of Medicine, National University of Malaysia (UKM), Kuala Lumpur Campus, Raja Muda Abdul Aziz Road, 50300 Kuala Lumpur, Malaysia; ^2^Department of Human Anatomy, Faculty of Medicine and Health Sciences, Putra University of Malaysia (UPM), 43400 UPM Serdang, Selangor, Malaysia

## Abstract

Oxidative stress and apoptosis can disrupt the bone formation activity of osteoblasts which can lead to osteoporosis. This study was conducted to investigate the effects of **γ**-tocotrienol on lipid peroxidation, antioxidant enzymes activities, and apoptosis of osteoblast exposed to hydrogen peroxide (H_2_O_2_). Osteoblasts were treated with 1, 10, and 100 **μ**M of **γ**-tocotrienol for 24 hours before being exposed to 490 **μ**M (IC_50_) H_2_O_2_ for 2 hours. Results showed that **γ**-tocotrienol prevented the malondialdehyde (MDA) elevation induced by H_2_O_2_ in a dose-dependent manner. As for the antioxidant enzymes assays, all doses of **γ**-tocotrienol were able to prevent the reduction in SOD and CAT activities, but only the dose of 1 **μ**M of GTT was able to prevent the reduction in GPx. As for the apoptosis assays, **γ**-tocotrienol was able to reduce apoptosis at the dose of 1 and 10 **μ**M. However, the dose of 100 **μ**M of **γ**-tocotrienol induced an even higher apoptosis than H_2_O_2_. In conclusion, low doses of **γ**-tocotrienol offered protection for osteoblasts against H_2_O_2_ toxicity, but itself caused toxicity at the high doses.

## 1. Introduction

Bone is a dynamic organ that carries out major functions of the body, which include maintenance of the mechanical integrity, body support, and regulation of mineral homeostasis. Bone is continually being resorped by osteoclasts and formed by osteoblasts to maintain bone volume and calcium and phosphorus homeostasis. The balance between bone formation and resorption is known as bone remodeling. If the balance is disturbed, the volume and quality of bone will be adversely affected, as in the case of osteoporosis.

Many studies and lines of evidence have linked oxidative stress to the pathogenesis of osteoporosis. Basu et al. [1] reported that there was a biochemical link between increased oxidative stress and decreased bone mineral density (BMD) in aged men and women. Maggio et al. [2] found that there was a significant decrease of plasma antioxidant levels for elderly women who have osteoporosis. Lean et al. [3] found that the thiol antioxidants in osteoclasts were lowered during estrogen deficiency. Oxidative stress may lead to bone loss by promoting lipid peroxidation [4, 5], lowering antioxidant enzymes [5], and promoting apoptosis of osteoblasts [6]. Several osteoporosis risk factors, such as smoking [7], hypertension [8], and diabetes mellitus [9], were related to oxidative stress.

Osteoblasts are important cells that are responsible for bone formation. Any reduction in the number or function of these cells to synthesize new bone matrix may result in osteoporosis [10, 11]. Several studies have shown that free radicals and reactive oxygen species (ROS) can affect the growth and function of these cells. Mody et al. [12] and Mogi et al. [13] showed that osteoblasts can produce ROS such as nitrogen oxide (NO) and hydrogen peroxide (H_2_O_2_) in response to inflammatory cytokines. These ROS may initiate lipid peroxidation [14], reduce antioxidant enzymes [15], and induce osteoblast apoptosis [16, 17]. These may adversely affect osteoblast numbers at bone formation site [18] and may contribute to bone loss [18, 19].

There is now a tendency towards the application of antioxidants in the protection and treatment of oxidative stress-related diseases. Vitamin E is a powerful biological antioxidant [20] with the ability to protect bone cells from the damages caused by lipid peroxidation [21]. Tocotrienols, the minor isomers of vitamin E, have gained scientific interest with the recent reports that they have better therapeutic potential than tocopherols [22]. Tocotrienols are the main constituent of vitamin E in palm oil *Elaeis guineensis,* and palm oil is the best source of tocotrienols, with 800 mg of tocotrienols for every kilogram of the crude oil [23]. *γ*-tocotrienol is the most abundant isomer in palm oil, making up 49% of vitamin E [24].

In bone studies, when the two types of vitamin E were compared in animal osteoporosis models, tocotrienol isomers were found to have better bone-protective effects than *α*-tocopherol. Norazlina et al. [25] have shown that tocotrienols were able to reverse bone loss induced by nicotine in rats. Palm oil-derived tocotrienols have also shown potential as prophylactic agents in prevention of glucocorticoid-induced osteoporosis in adrenalectomized rat [26]. The bone-protective mechanism of vitamin E was thought to be contributed by its antioxidant property. This was confirmed by a study which found that vitamin E especially tocotrienols protected rat bones against damage caused by free radicals released by an oxidizing agent [27]. Hermizi et al. [28] showed that *γ*-tocotrienol not only reversed nicotine-induced osteoporosis better than tocopherol, but also improved the bone structure until it was better than the normal control rats. This has led to a study which confirmed that vitamin E, especially tocotrienols, has bone anabolic effects on normal male rats [29]. Tocotrienols were also found to be better than tocopherol in improving the static and dynamic bone histomorphometric parameters [30]. The most recent study found that *α*-tocotrienol, but not *α*-tocopherol, prevented osteoclastic bone resorption by inhibiting RANKL expression and blocking RANKL action on osteoclast precursors [31].

Although *in vivo* studies showed that tocotrienols exhibit bone-protective activity, there is paucity of *in vitro* studies to determine the effect of tocotrienols on bone cells. Low doses of *γ*-tocotrienol were found to be better than *α*-tocopherol in protecting rat osteoblasts against H_2_O_2_ toxicity. However, higher doses of *γ*-tocotrienol were found to be toxic to rat osteoblasts [28]. This paradoxical effect of *γ*-tocotrienol needs further investigation on how the protective effects were not only lost at high dose of *γ*-tocotrienol, but it became toxic to osteoblasts.

 It was suggested that at high dose, tocotrienol may become pro-oxidant or proapoptotic, which may be responsible for its toxic effects on osteoblasts. In order to confirm this, the study was focused on determining the effects of low and high doses of *γ*-tocotrienol on the index of lipid peroxidation and apoptosis of osteoblasts.

## 2. Materials and Methods

### 2.1. Culture of Osteoblasts

Osteoblasts were isolated using the explant culture method [32]. Briefly, Sprague-Dawley male rats (after weaning, 4–6 weeks old, weight 40–60 g) were sterilely dissected, and the long bones (femur, tibia, fibula, radius, and ulna) were collected and scraped until cleaned from the remaining muscle and connective tissues. The bones were cut into small pieces (1-2 mm) and sterilized in 50 *μ*g/mL gentamycin (Sigma) in PBS. The bone pieces were then digested with collagenase solution (type IA, Sigma) (2 mg/mL in DMEM) for 2 hours in shaking water bath (37°C, 150 rpm) to remove the remaining soft tissues. The bone pieces were then rinsed with PBS before plated into 25 mm^2^ flask containing 5 mL DMEM (10% FCS, 50 *μ*g/mL gentamycin) and incubated in CO_2_ incubator (37°C, 5%  CO_2_) until confluence. This study was approved by the Universiti Kebangsaan Malaysia Animal Ethic Committee (UKMAEC) with the approval number FAR/2006/NAZRUN/24-JULY/171-JANUARY-2007.

### 2.2. Treatment of Osteoblasts

Osteoblast number was prepared at 1 × 10^7^ cells for measurement of MDA levels, 2 × 10^6^ cells for measurements of glutathione peroxidase, superoxide dismutase, catalase, and caspase-3 enzymes activities, and 2 × 10^5^ cells for single-stranded DNA analysis. Osteoblasts were incubated in CO_2_ incubator (37°C, 5%  CO_2_) with 1, 10, and 100 *μ*M of *γ*-tocotrienol extracted from palm oil (Carotech, Malaysia) for 24 hours before incubated with H_2_O_2_. The incubation period with H_2_O_2_ was 2 hours at the concentration of 490 *μ*M, which was the IC_50_ of H_2_O_2_ [32].

The doses of *γ*-tocotrienol used were based on previous study. These doses were able to cover both spectrums of *γ*-tocotrienol activities at low and high doses [28]. Every concentration was repeated triplicate, using 3 different osteoblast cultures.

### 2.3. MDA Levels

The MDA level was measured using Biotech LPO-586 (OxisResearch, US) based on the reaction of a chromogenic reagent, N-methyl-2-phenylindole, with MDA at 45°C which yields a stable chromophore that can be measured at the absorbance of 586 nm. Briefly, 1 mL of cell supernatants that were obtained by scraping, sonicating, and centrifugation (3000 ×g, 10 min) of cells monolayer (1 × 10^7^ cells) in cold environment (4°C) was mixed properly with 650 *μ*L R1 reagent (N-methyl-2-phenylindole in acetonitrile diluted 3 times with ferum ion solution in methanol) and 150 *μ*L concentrated HCl (12N, 37%). The samples were then heated in water bath (45°C, 60 min) before centrifuged (15 000 ×g, 10 min) to obtain the supernatants that were measured spectrometrically (586 nm).

### 2.4. Glutathione Peroxidase Activity

Glutathione peroxidase (GPx) activity was measured using the Glutathione Peroxidase Assay Kit (Cayman Chemical, US). The kit measures GPx activity indirectly by a coupled reaction with glutathione reductase (GR). Oxidized glutathione (GSSG), produced upon reduction of hydroperoxide by GPx, was recycled to its reduced state by GR and NADPH. The oxidation of NADPH to NADP^+^ is accompanied by a decrease in absorbance at 340 nm. The rate of decrease in the absorbance is directly proportional to the GPx activity in the sample. Briefly, 20 *μ*l of cell supernatants that were obtained by scraping, sonicating, and centrifugation (10 000 ×g, 15 min) of cells monolayer (2 × 10^6^ cells) in cold environment (4°C) was added with 100 *μ*L assay buffer (50 mM Tris-HCl, pH 7.6 contains 5 mM EDTA) and 50 *μ*L cosubstrate mixture (NADPH, glutathione, and glutathione reductase) in a 96-well plate. The reaction was started by adding 20 *μ*L cumene hydroperoxide and the absorbance (340 nm) measured kinetically every minute for 5 minutes by using ELISA reader (Versamax, US). GPx activity was calculated by using the formula

(1) GPx  activity=[(ΔA340min⁡0.00373 μM−1)×(0.19 mL0.02 mL  )×sample  dilution  factor],

where Δ_A340min⁡_ was the difference of absorbance calculated by using the formula

(2) ΔA340min⁡  =(Absorbance  at  time  A−Absorbance  at  time  B)(time  A−time  B).

GPx activity was stated in nmol/min/mL by assuming that 1 unit of enzyme oxidizes 1 nmol of NADPH to NADP^+^ at 25°C.

### 2.5. Superoxide Dismutase Activity

The superoxide dismutase (SOD) activity was measured using the Superoxide Dismutase Assay Kit (Cayman Chemical, US). The kit utilizes a tetrazolium salt for detection of superoxide radicals generated by xanthine oxidase and hypoxanthine. One unit of SOD is defined as the amount of enzyme needed to exhibit 50% dismutation of the superoxide radical. Briefly, 10 *μ*l of cell supernatants that were obtained by scraping, sonicating, and centrifugation (15 000 ×g, 5 min) of cells monolayer (2 × 10^6^ cells) in cold environment (4°C) was added to 200 *μ*L radical detector (50 *μ*L tetrazolium mixed with 19.95 mL assay buffer, i.e., 50 mM Tris-HCl, pH 8.0 contained 0.1 mM DTPA and 0.1 mM hypoxanthine). The reaction was started by adding 20 *μ*L of xanthine oxide in a 96-well plate. The plate was gently shaken and incubated (20 min, room temperature) before the absorbance (450 nm) measured by using ELISA reader (Versamax, US). Standard curve of linearize rate (LR) of absorbance versus SOD activities was plotted, and the SOD activities in the samples were calculated by using the formula

(3) SOD(UmL)={([LR  sample−y-intercept]slope)×(0.23 mL0.01 mL  )}×sample  dilution  factor.

The linearized rate (LR) was calculated by dividing all the absorbance values with standard absorbance value (SOD 0.0 U/mL).

### 2.6. Catalase Activity

The catalase (CAT) activity was measured using the Catalase Assay Kit (Cayman Chemical, US). The method was based on the reaction of CAT with methanol in the presence of H_2_O_2_. The formaldehyde produced was then measured chromatically (450 nm) with 4-amino-3-hydrazino-5-mercapto-l,2,4-triazole as the chromogen. Briefly, 20 *μ*l of cell supernatants that were obtained by scraping, sonicating, and centrifugation (10 000 ×g, 15 min) of cells monolayer (2 × 10^6^ cells) in cold environment (4°C) was added with 100 *μ*L assay buffer (100 mM potassium phosphate, pH 7.0) and 30 *μ*L methanol in a 96-well plate. The standard was prepared by mixing 100 *μ*L assay buffer with 30 *μ*L methanol and 20 *μ*L formaldehyde (0, 5, 15, 30, 45, 60, and 75 *μ*M). The reaction was started by adding 20 *μ*L diluted H_2_O_2_ (40 *μ*L H_2_O_2_ with 9.96 mL HPLC-grade water) into all wells. The plate was then incubated for 20 minutes at room temperature on a shaker. The reaction was stopped by adding 30 *μ*L KOH 0.5 M and 30 *μ*L chromogen, and the plate was measured spectrometrically (540 nm) using ELISA reader (Versamax, US). The standard curve of absorbance versus formaldehyde concentrations was plotted, and formaldehyde concentration in the samples was calculated by using the formula

(4) Formaldehyde  concentration  (μM)  =([sample  absorbance−y-intercept]slope)   ×(0.19 mL0.02 mL).

CAT activity was expressed in nmol/min/mL by assuming that 1 unit of enzyme produces 1 nmol of formaldehyde at 25°C.

### 2.7. Caspase-3 Activity

The caspase-3 activity was measured using CaspASE Assay System Colorimetric Kit (Promega, US). Briefly, 20 *μ*L of cell supernatants was obtained by scraping, lysing with lysis buffer and free-thaw cycles, and centrifugation (15 000 ×g, 20 min, 4°C) of the monolayer of the cells (2 × 10^6^ cells). 32 *μ*L caspase buffer, 2 *μ*L DMSO, 10 *μ*L DTT, and 78 *μ*L deionized water were added into a 96-well plate. The reaction was started by adding 2 *μ*L DEVD-pNA substrate and incubated (37°C, 4 h). The absorbances of the samples were measured spectrometrically (405 nm).

### 2.8. Single-Stranded DNA Analysis

The single-stranded DNA (ssDNA) was analyzed using ssDNA Apoptosis ELISA Kit (Chemicon, US). This procedure was based on selective DNA denaturation in apoptotic cells by formamide and detection of the denatured DNA by monoclonal antibody to single-stranded DNA. Briefly, the cells in a 96-well plate were fixed with 80% methanol in PBS before treated by formamide and denaturized by heating (75°C, 10 min) and cooling (4°C, 5 min). Negative control was prepared by adding 100 unit/mL SI nuclease and incubated (37°C, 1 hour), while positive control was prepared by adding 100 *μ*L ssDNA solution. All wells were dried overnight before washed 3 times with PBS. After blocking the nonspecific sites with 200 *μ*L nonfat milk 3% for 1 h, all wells were added with antibody mixture and incubated for 30 minutes before washed 3 times with PBS. ABTS solution was added, and the absorbance was read at 405 nm (Versamax, US) after incubation of 60 min.

### 2.9. Protein Content Determination

Protein content determination used in the analysis of MDA levels and GPx activity was measured by the method of Bradford [33].

### 2.10. Statistical Analysis

Every concentration was repeated triplicate and using 3 different osteoblast cultures with comparable results. All data were analyzed by one-way ANOVA by using SPSS version 13 software and expressed in mean ± standard deviation. *P* < 0.05 was considered significant.

## 3. Results

### 3.1. MDA Levels

**Figure 1:**
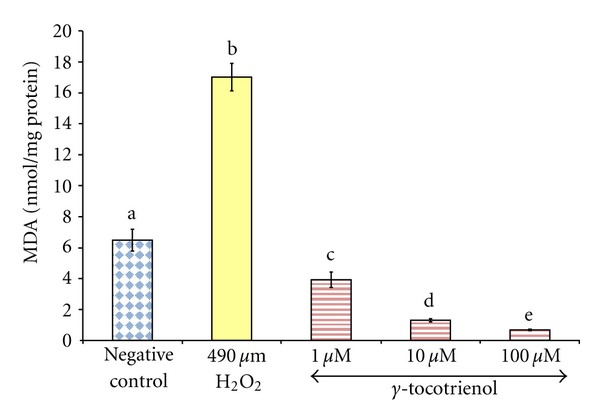
The effects of H_2_O_2_ and *γ*-tocotrienol on the MDA levels in osteoblasts. Osteoblasts were pretreated with 1, 10, and 100 *μ*M *γ*-tocotrienol for 24 hours before treated with 490 *μ*M H_2_O_2_ for 2 hours. The groups that have the same alphabet symbols (a, b, c, d, e) are not significantly different from each other (*P* < 0.05). Data are presented as mean ± SEM, *n* = 3.

Exposure of osteoblasts to 490 *μ*M H_2_O_2_ for 2 hours significantly increased the MDA levels compared to the control group. Pretreatments with *γ*-tocotrienol prevented MDA elevation induced by H_2_O_2_ in a dose-dependent manner (Figure 1).

### 3.2. GPx Activity

**Figure 2:**
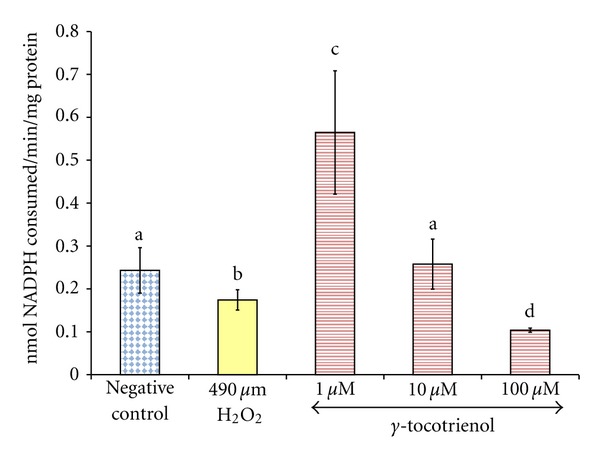
The effects of H_2_O_2_ and *γ*-tocotrienol on the GPx activity in osteoblasts. Osteoblasts were pretreated with 1, 10, and 100 *μ*M *γ*-tocotrienol for 24 hours before treated with 490 *μ*M H_2_O_2_ for 2 hours. The groups that have the same alphabet symbols (a, b, c, d) are not significantly different from each other (*P* < 0.05). Data are presented as mean ± SEM, *n* = 3.

Exposure of osteoblasts to 490 *μ*M H_2_O_2_ for 2 hours significantly reduced the GPx activity compared to the control group. The group pretreated with 1 *μ*M *γ*-tocotrienol had the highest GPx activity compared to other groups, while the pretreatment with 10 and 100 *μ*M *γ*-tocotrienol did not prevent the reduction in GPx activity induced by H_2_O_2_. The group pretreated with 100 *μ*M *γ*-tocotrienol also had the lowest GPx activity compared to other groups (Figure 2).

### 3.3. SOD Activity

**Figure 3:**
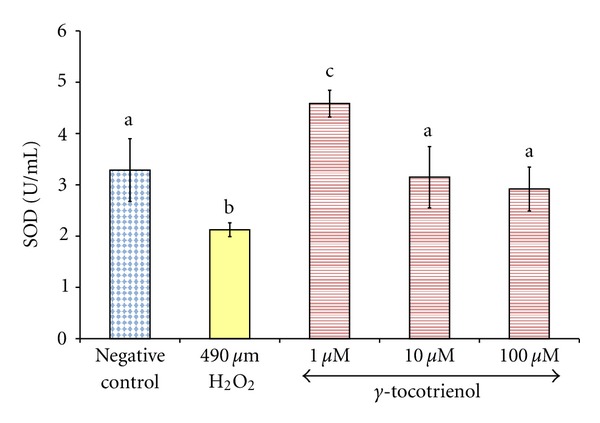
The effects of H_2_O_2_ and *γ*-tocotrienol on the SOD activity in osteoblasts. Osteoblasts were pretreated with 1, 10, and 100 *μ*M *γ*-tocotrienol for 24 hours before treated with 490 *μ*M H_2_O_2_ for 2 hours. The groups that have the same alphabet symbols (a, b, c) are not significantly different from each other (*P* < 0.05). Data are presented as mean ± SEM, *n* = 3.

Exposure of osteoblasts to 490 *μ*M H_2_O_2_ for 2 hours significantly reduced the SOD activity compared to the control group. Pretreatment with *γ*-tocotrienol at doses 1, 10, and 100 *μ*M for 24 hours had prevented the reduction of SOD activity induced by H_2_O_2_ (Figure 3).

### 3.4. CAT Activity

**Figure 4:**
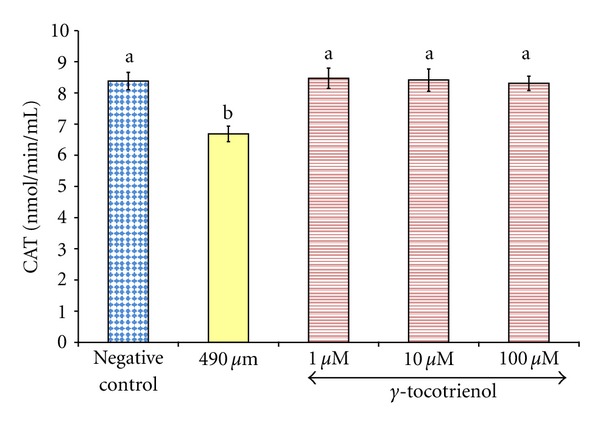
The effects of H_2_O_2_ and *γ*-tocotrienol on the CAT activity in osteoblasts. Osteoblasts were pretreated with 1, 10, and 100 *μ*M *γ*-tocotrienol for 24 hours before treated with 490 *μ*M H_2_O_2_ for 2 hours. The groups that have the same alphabet symbols (a, b) are not significantly different from each other (*P* < 0.05). Data are presented as mean ± SEM, *n* = 3.

Exposure of osteoblasts to 490 *μ*M H_2_O_2_ for 2 hours significantly reduced the CAT activity compared to the control group. Pretreatment with *γ*-tocotrienol at doses 1, 10, and 100 *μ*M for 24 hours had prevented the reduction of CAT activity induced by H_2_O_2_ (Figure 4).

### 3.5. Caspase-3 Activity

**Figure 5:**
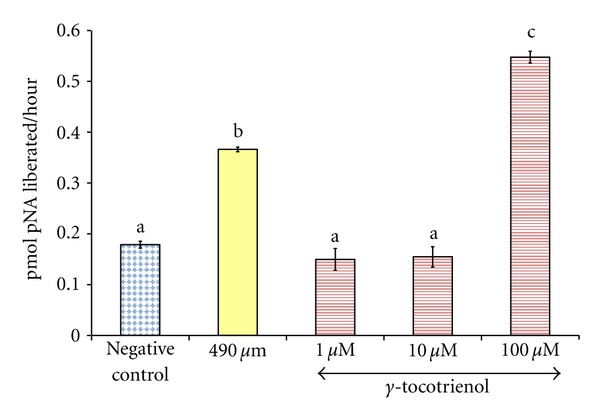
The effects of H_2_O_2_ and *γ*-tocotrienol on the caspase-3 activity in osteoblasts. Osteoblasts were pretreated with 1, 10, and 100 *μ*M *γ*-tocotrienol for 24 hours before treated with 490 *μ*M H_2_O_2_ for 2 hours. The groups that have the same alphabet symbols (a, b, c) are not significantly different from each other (*P* < 0.05). Data are presented as mean ± SEM, *n* = 3.

Exposure of osteoblasts to 490 *μ*M H_2_O_2_ for 2 hours significantly increased the caspase-3 activity in the cells compared to the control group. Pretreatment with 1 and 10 *μ*M of *γ*-tocotrienol prevented the increase in caspase-3 activity induced by H_2_O_2_, but pretreatment with 100 *μ*M resulted in the highest caspase-3 activity compared to other groups (Figure 5).

### 3.6. ssDNA Analysis

**Figure 6:**
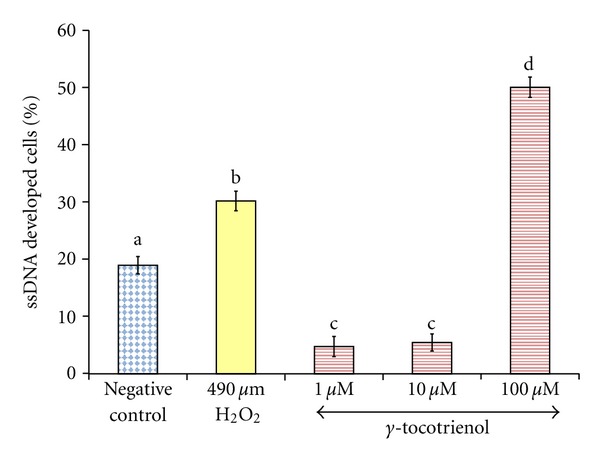
The effects of H_2_O_2_ and *γ*-tocotrienol on the percentage of ssDNA developed in osteoblasts. Osteoblasts were pretreated with 1, 10, and 100 *μ*M *γ*-tocotrienol for 24 hours before treated with 490 *μ*M H_2_O_2_ for 2 hours. The groups that have the same alphabet symbols (a, b, c, d) are not significantly different between each other (*P* < 0.05). Data are presented as mean ± SEM, *n* = 3.

Exposure of osteoblasts to 490 *μ*M H_2_O_2_ for 2 hours significantly increased the ssDNA levels in the cells compared to the control group. Pretreatment with 1 and 10 *μ*M of *γ*-tocotrienol for 24 hours significantly reduced ssDNA levels when compared to the control group and H_2_O_2_ groups. However, pretreatment of 100 *μ*M *γ*-tocotrienol resulted in the highest ssDNA level compared to other groups (Figure 6).

## 4. Discussion

Lipid peroxidation is closely associated with osteoporosis. Parhami et al. [4] showed that the lipids that accumulated in human osteoporotic bones were oxidized and become hazardous to the bone cells. The lipids accumulation and oxidation may reverse the normal control of the local biomineralization process, by encouraging calcification in soft tissue and osteolysis [34]. Oxidized lipids promoted bone resorption [35] by promoting recruitment and differentiation of osteoclast precursor and inhibition of osteoblasts differentiation [36]. The present study showed that the MDA level of osteoblasts exposed to H_2_O_2_ was elevated. Similar increase in the MDA levels was reported in MC3T3-E1 preosteoblast cell line [14] and bone marrow stromal cells [37] when exposed to H_2_O_2_. Both the H_2_O_2_ and lipid peroxidation levels were reported to be elevated in the femoral tissue homogenate of ovariectomized rats [38].

In the present study, pretreatment with *γ*-tocotrienol prevented the MDA elevation of osteoblasts exposed to H_2_O_2_ in dose-dependent manner. The antilipid peroxidation property of vitamin E is contributed by the phenolic hydroxyl group of vitamin E which easily donates hydrogen atoms to the peroxyl radical, thus creating a more stable lipid species. The efficiency of this protective mechanism is dependent on the mobility of vitamin E in the membrane and its ability to contribute electrons, which is determined by the aliphatic side chains and the number of methyl groups on the chromanoxyl ring, respectively [39].

Tocotrienols were reported to exhibit antilipid peroxidation activity in liver cells [40], brain cells [41], red blood cells [42], and low-density lipoprotein (LDL) [43]. Tocotrienol was better than tocopherol in protecting HUVEC cells exposed to arachidonic acid [43] and RAT-1 fibroblasts exposed to H_2_O_2_ [44]. In an *in vivo* study, Maniam et al. [45] found that the femoral bone TBARS levels decreased dose dependently with palm tocotrienol supplementation.

Serbinova et al. [46] suggested that tocotrienol has superior antioxidant activity than tocopherol due to the more uniform distribution in the membrane bilayers and higher displacement of the membrane lipids. Palozza et al. [44] hypothesized that the unsaturated double bonds of tocotrienol enable trapping of radicals in both hydrophilic and lipophilic compartment, facilitating its absorption [47] and mobility [44] in the cell membrane.

The present study found that H_2_O_2_ significantly reduced the GPx, SOD, and CAT activities, indicating disruptions of the endogenous antioxidant enzymes. Similar findings were demonstrated with pheochromocytoma cell lines (PC12) [48], HUVEC cells [49], rat hepatocytes [50], bone marrow stromal cells [37], and MC3T3-E1 cells [51]. The antioxidant enzyme was used up to eliminate the lipid peroxidation products or deactivated and glycated by the radicals [52, 53]. Therefore, exogenous antioxidants such as vitamin E are required to assist endogenous antioxidants in eliminating free radicals and reactive species. Vitamin E is also involved in the glutathione redox cycle which allows glutathione to be regenerated [54, 55].

In the present study, all doses of *γ*-tocotrienol prevented the reductions in osteoblastic SOD and CAT activities, but only the dose of 1 *μ*M of *γ*-tocotrienol prevented the reduction in GPx activity. The restoration of the antioxidant enzymes activities may be contributed by the ability of vitamin E to elevate the mRNA expression of these enzymes under stressful condition [56]. Another possibility is that vitamin E may have stabilized the mRNA of antioxidant enzymes after transcription process and enhanced the translation of the derived enzyme proteins [57]. The antioxidant enzymes expression may have been modulated by vitamin E via peroxisome proliferator-activated receptors *γ* (PPAR*γ*) and nuclear factor-kappaB (NF-*κ*B) [58].

Unexpectedly, there was a paradoxical reduction in the GPx activity with the high dose of *γ*-tocotrienol. Antioxidants can become pro-oxidants at certain concentrations or with the presence of oxygen or metal ions [59]. Mazlan et al. [60] suggested that at high concentrations, *γ*-tocotrienol turns to pro-oxidant and causes toxicity to astrocytes. Low concentration (0.3 mM) of vitamin C induced the differentiation of preosteoblasts (MC3T3-E1) [61], but at high concentration (1 mM), it increased oxidative stress, reduced viability, and caused morphological changes in lung endothelial cells [62]. Other antioxidants were also reported to become pro-oxidant at high concentration such as *β*-carotene [63], amyloid *β*-peptide [64], and vitamin A [65, 66]. The presence of transition metal ions may cause an antioxidant to become pro-oxidant as in the case of *α*-tocopherol [67], amyloid *β*-peptide [68], and vitamin C [58]. However, this was unlikely without the presence of transition metal ions in the present study.

The present study found that the activation of the caspase-3 activity may have caused apoptosis of the osteoblasts exposed to H_2_O_2_. This was consistent with a study which found elevation of caspase-3 activity in preosteoblast cell lines (MC3T3-E1) exposed to H_2_O_2_ [69]. The apoptosis and caspase-3 activities were found to be elevated in human vascular endothelial cells (ECU-304), bone marrow stromal cells, and HUVEC exposed to H_2_O_2_ [37, 49, 70]. Caspase-3 is the main executor of the caspase group that led to the pathway of apoptosis [71]. Caspase-3 induces apoptosis by cleaving DNA repair molecules, degrading antiapoptotic protein, and cleaving the extracellular matrix protein, skeleton proteins, and related molecules [72]. In the present study, the osteoblast apoptosis by H_2_O_2_ was associated with the denaturation of osteoblast DNA. H_2_O_2_ was found to adversely affect the DNA of MC3T3-E1 cells through the inhibition of DNA synthesis [73], DNA fragmentation [69], and nuclei condensation [51], which are characteristics of apoptosis. It was also reported to inhibit osteogenic differentiation, increase the ROS levels, activate the caspase activity, and eventually induce apoptosis [74–76].

Agents that inhibit the production of reactive oxygen species or increase the antioxidant defense may prevent apoptosis and protect cells from oxygen radicals damage [77–79]. In the present study, low concentration of *γ*-tocotrienol was able to protect osteoblasts from H_2_O_2_ induced apoptosis, but *α*-tocopherol was not able to do so. This was consistent with studies which found that low concentrations of *γ*-tocotrienol (1 and 10 *μ*M) were able to protect rat primary astrocytes [60], rat primary cerebellar cells [80], rat primary cortical neuronal cells, and SH-SY5Y cells [81] from H_2_O_2_-induced apoptosis. Paradoxically, higher concentration of *γ*-tocotrienol (100 *μ*M) was found to promote osteoblast apoptosis. This was reflected with the excessively high caspace-3 activity of osteoblast treated with 100 *μ*M of *γ*-tocotrienol. According to Then et al. [80], H_2_O_2_ activated both the intrinsic pathway through caspase-9 and extrinsic pathway through caspase-8 before they activate caspase-3. The present study confirmed that *γ*-tocotrienol had caused apoptosis via activation of caspace-3, but the actual pathway is not well understood [82].

There is still a question regarding the cause of tocotrienol to become proapoptotic at high concentration. Birringer et al. [83] found that HepG2 cells metabolized tocopherols and tocotrienols to short- and long-chain metabolites, with greater tocotrienol metabolites being produced. Recently, a study has found that the tocopherol metabolite of long chain (13′-carboxychromanol) was a strong inducer of apoptosis [84]. Although there was no studies done to confirm this, tocotrienol metabolites may have contributed to the proapoptotic effect of tocotrienol. At low concentrations, *γ*-tocotrienol prevented apoptosis by increasing the endogenous antioxidant capacity, reducing lipid peroxidation, inhibiting the apoptosis pathway, and reducing the DNA fragmentation. Nanomolar concentrations of tocotrienol have been found to inhibit apoptosis pathway signals including src kinase [85, 86] and 12-lipoxygenase [87].

Based on the result of the present and previous studies, the toxic effects of *γ*-tocotrienol may have been contributed by its proapoptotic effects at higher doses. Although high dose of *γ*-tocotrienol reduced the glutathione peroxidase activity, the lipid peroxidation level was still suppressed. At low doses, *γ*-tocotrienol has potential to be used for the treatment and prevention of diseases related to oxidative stress including osteoporosis. However, at high doses, *γ*-tocotrienol may be toxic to cells by promoting apoptosis. This paradoxical effect of *γ*-tocotrienol at high doses may be useful for killing cancer cells.

In conclusion, low doses of *γ*-tocotrienol (1 and 10 *μ*M) offered osteoblasts protection against H_2_O_2_-induced oxidative stress and apoptosis. Paradoxically, high dose of *γ*-tocotrienol (100 *μ*M) reduced glutathione peroxidase activity and promoted apoptosis. Further studies are required to determine the exact apoptosis pathway involved and possible involvement of the tocotrienol metabolites.

## References

[1] Basu S, Michaëlsson K, Olofsson H, Johansson S, Melhus H (2001). Association between oxidative stress and bone mineral density. *Biochemical and Biophysical Research Communications*.

[2] Maggio D, Barabani M, Pierandrei M (2003). Marked decrease in plasma antioxidants in aged osteoporotic women: results of a cross-sectional study. *Journal of Clinical Endocrinology and Metabolism*.

[3] Lean JM, Davies JT, Fuller K (2003). A crucial role for thiol antioxidants in estrogen-deficiency bone loss. *Journal of Clinical Investigation*.

[4] Parhami F, Morrow AD, Balucan J (1997). Lipid oxidation products have opposite effects on calcifying vascular cell and bone cell differentiation: a possible explanation for the paradox of arterial calcification in osteoporotic patients. *Arteriosclerosis, Thrombosis, and Vascular Biology*.

[5] Ozgocmen S, Kaya H, Fadillioglu E, Aydogan R, Yilmaz Z (2007). Role of antioxidant systems, lipid peroxidation, and nitric oxide in postmenopausal osteoporosis. *Molecular and Cellular Biochemistry*.

[6] Manolagas SC (2008). De-fense! de-fense! de-fense: scavenging H_2_O_2_ while making cholesterol. *Endocrinology*.

[7] Law MR, Hackshaw AK (1997). A meta-analysis of cigarette smoking, bone mineral density and risk of hip fracture: recognition of a major effect. *British Medical Journal*.

[8] Cappuccio FP, Meilahn E, Zmuda JM, Cauley JA (1999). High blood pressure and bone-mineral loss in elderly white women: a prospective study. *The Lancet*.

[9] Christensen JO, Svendsen OL (1999). Bone mineral in pre- and postmenopausal women with insulin-dependent and non-insulin-dependent diabetes mellitus. *Osteoporosis International*.

[10] Nishida S, Endo N, Yamagiwa H, Tanizawa T, Takahashi HE (1999). Number of osteoprogenitor cells in human bone marrow markedly decreases after skeletal maturation. *Journal of Bone and Mineral Metabolism*.

[11] Arjmandi BH, Juma S, Beharka A, Bapna MS, Akhter M, Meydani SN (2002). Vitamin E improves bone quality in the aged but not in young adult male mice. *Journal of Nutritional Biochemistry*.

[12] Mody N, Parhami F, Sarafian TA, Demer LL (2001). Oxidative stress modulates osteoblastic differentiation of vascular and bone cells. *Free Radical Biology and Medicine*.

[13] Mogi M, Kinpara K, Kondo A, Togari A (1999). Involvement of nitric oxide and biopterin in proinflammatory cytokine-induced apoptotic cell death in mouse osteoblastic cell line MC3T3-E1. *Biochemical Pharmacology*.

[14] Choi EM, Kim GH, Lee YS (2009). Protective effects of dehydrocostus lactone against hydrogen peroxide-induced dysfunction and oxidative stress in osteoblastic MC3T3-E1 cells. *Toxicology in Vitro*.

[15] Xiao Y, Cui J, Shi Y, Le G (2012). Alpha-lipoic acid protects against hydrogen peroxide-induced oxidative stress in MC3T3-E1 osteoblast-like cells. *Journal of Functional Foods*.

[16] Chen RM, Liu HC, Lin YL, Jean WC, Chen JS, Wang JH (2002). Nitric oxide induces osteoblast apoptosis through the de novo synthesis of Bax protein. *Journal of Orthopaedic Research*.

[17] Fatokun AA, Stone TW, Smith RA (2006). Hydrogen peroxide-induced oxidative stress in MC3T3-E1 cells: the effects of glutamate and protection by purines. *Bone*.

[18] Weinstein RS, Jilka RL, Parfitt AM, Manolagas SC (1998). Inhibition of osteoblastogenesis and promotion of apoptosis of osteoblasts end osteocytes by glucocorticoids potential mechanisms of their deleterious effects on bone. *Journal of Clinical Investigation*.

[19] Jilka RL, Weinstein RS, Bellido T, Parfitt AM, Manolagas SC (1998). Osteoblast programmed cell death (apoptosis): modulation by growth factors and cytokines. *Journal of Bone and Mineral Research*.

[20] Crary EJ, McCarty MF (1984). Potential clinical applications for high-dose nutritional antioxidants. *Medical Hypotheses*.

[21] Xu H, Watkins BA, Seifert MF (1995). Vitamin E stimulates trabecular bone formation and alters epiphyseal cartilage morphometry. *Calcified Tissue International*.

[22] Schaffer S, Müller WE, Eckert GP (2005). Tocotrienols: constitutional effects in aging and disease. *Journal of Nutrition*.

[23] Sen CK, Khanna S, Roy S (2006). Tocotrienols: vitamin E beyond tocopherols. *Life Sciences*.

[24] Puah CW, Choo YM, Ma AN, Chuah CH (2007). The effect of physical refining on palm vitamin E (tocopherol, tocotrienol and tocomonoenol). *American Journal of Applied Sciences*.

[25] Norazlina M, Lee PL, Lukman HI, Nazrun AS, Ima-Nirwana S (2007). Effects of vitamin E supplementation on bone metabolism in nicotine-treated rats. *Singapore Medical Journal*.

[26] Ima-Nirwana S, Kiftiah A, Sariza T, Gapor MTA, Khalid BAK (1999). Palm vitamin E improves bone metabolism and survival rate in thyrotoxic rats. *General Pharmacology*.

[27] Ahmad NS, Khalid BAK, Luke DA, Nirwana SI (2005). Tocotrienol offers better protection than tocopherol from free radical-induced damage of rat bone. *Clinical and Experimental Pharmacology and Physiology*.

[28] Hermizi H, Faizah O, Ima-Nirwana S, Ahmad Nazrun S, Norazlina M (2009). Beneficial effects of tocotrienol and tocopherol on bone histomorphometric parameters in Sprague-Dawley male rats after nicotine cessation. *Calcified Tissue International*.

[29] Shuid AN, Mehat Z, Mohamed N, Muhammad N, Soelaiman IN (2010). Vitamin E exhibits bone anabolic actions in normal male rats. *Journal of Bone and Mineral Metabolism*.

[30] Mehat MZ, Shuid AN, Mohamed N, Muhammad N, Soelaiman IN (2010). Beneficial effects of vitamin e isomer supplementation on static and dynamic bone histomorphometry parameters in normal male rats. *Journal of Bone and Mineral Metabolism*.

[31] Ha H, Lee JH, Kim HN, Lee ZH (2011). *α*-Tocotrienol inhibits osteoclastic bone resorption by suppressing RANKL expression and signaling and bone resorbing activity. *Biochemical and Biophysical Research Communications*.

[32] Nizar AM, Nazrun AS, Norazlina M, Norliza M, Ima Nirwana S (2011). Low dose of tocotrienols protects osteoblasts against oxidative stress. *La Clinica Terapeutica*.

[33] Bradford MM (1976). A rapid and sensitive method for the quantitation of microgram quantities of protein utilizing the principle of protein dye binding. *Analytical Biochemistry*.

[34] Demer LL (2002). Vascular calcification and osteoporosis: inflammatory responses to oxidized lipids. *International Journal of Epidemiology*.

[35] Garrett IR, Boyce BF, Oreffo ROC, Bonewald L, Poser J, Mundy GR (1990). Oxygen-derived free radicals stimulate osteoclastic bone resorption in rodent bone in vitro and in vivo. *Journal of Clinical Investigation*.

[36] Parhami F, Garfinkel A, Demer LL (2000). Role of lipids in osteoporosis. *Arteriosclerosis, Thrombosis, and Vascular Biology*.

[37] Qiang H, Gao P, Zhang C (2009). Effects of Panax notoginseng saponins on apoptosis induced by hydrogen peroxide in cultured rabbit bone marrow stromal cells via altering the oxidative stress level and down-regulating caspase-3. *Journal of Nanjing Medical University*.

[38] Muthusami S, Ramachandran I, Muthusamy B (2005). Ovariectomy induces oxidative stress and impairs bone antioxidant system in adult rats. *Clinica Chimica Acta*.

[39] Suzuki YJ, Tsuchiya M, Wassall SR (1993). Structural and dynamic membrane properties of *α*-tocopherol and *α*- tocotrienol: implication to the molecular mechanism of their antioxidant potency. *Biochemistry*.

[40] Kamat JP, Sarma HD, Devasagayam TRA, Nesaretnam K, Basiron Y (1997). Tocotrienols from palm oil as effective inhibitors of protein oxidation and lipid peroxidation in rat liver microsomes. *Molecular and Cellular Biochemistry*.

[41] Kamat JP, Devasagayam TPA (1995). Tocotrienols from palm oil as potent inhibitors of lipid peroxidation and protein oxidation in rat brain mitochondria. *Neuroscience Letters*.

[42] Tatsuta T (1971). Relationship between chemical structure and biological activity of vitamin E. I. Free tocopherols. *Vitamins*.

[43] Mutalib MSA, Khaza’ai H, Wahle KWJ (2003). Palm-tocotrienol rich fraction (TRF) is a more effective inhibitor of LDL oxidation and endothelial cell lipid peroxidation than *α*-tocopherol in vitro. *Food Research International*.

[44] Palozza P, Simone R, Picci N (2008). Design, synthesis, and antioxidant potency of novel *α*-tocopherol analogues in isolated membranes and intact cells. *Free Radical Biology and Medicine*.

[45] Maniam S, Mohamed N, Shuid AN, Soelaiman IN (2008). Palm tocotrienol exerted better antioxidant activities in bone than *α*-tocopherol. *Basic and Clinical Pharmacology and Toxicology*.

[46] Serbinova E, Kagan V, Han D, Packer L (1991). Free radical recycling and intramembrane mobility in the antioxidant properties of *α*-tocopherol and *α*-tocotrienol. *Free Radical Biology and Medicine*.

[47] Yoshida Y, Niki E, Noguchi N (2003). Comparative study on the action of tocopherols and tocotrienols as antioxidant: chemical and physical effects. *Chemistry and Physics of Lipids*.

[48] Wu JH, Xu C, Shan CY, Tan RX (2006). Antioxidant properties and PC12 cell protective effects of APS-1, a polysaccharide from *Aloe vera* var.* chinensis*. *Life Sciences*.

[49] Wang YK, Hong YJ, Wei M (2010). Curculigoside attenuates human umbilical vein endothelial cell injury induced by H_2_O_2_. *Journal of Ethnopharmacology*.

[50] Kim KW, Suh SJ, Kim JD (2009). Effects on lipid peroxidation and antioxidative enzymes of *Euonymus alatus* in cultured rat hepatocytes. *Basic and Clinical Pharmacology and Toxicology*.

[51] Xu ZS, Wang XY, Xiao DM (2011). Hydrogen sulfide protects MC3T3-E1 osteoblastic cells against H_2_O_2_-induced oxidative damage-implications for the treatment of osteoporosis. *Free Radical Biology and Medicine*.

[52] Manju V, Nalini N (2005). Chemopreventive efficacy of ginger, a naturally occurring anticarcinogen during the initiation, post-initiation stages of 1,2 dimethylhydrazine-induced colon cancer. *Clinica Chimica Acta*.

[53] Hodgson EK, Fridovich I (1975). The interaction of bovine erythrocyte superoxide dismutase with hydrogen peroxide: inactivation of the enzyme. *Biochemistry*.

[54] Parker L (1991). Vitamin E is nature’s master antioxidants. *Science and Medicine*.

[55] de Mulder CL, Madabushi HT, Tappel AL (1995). Protection by vitamin E, selenium, trolox, ascorbic acid palmitate, acetylcystine, coenzyme Q, beta-carotene, and (+)-catechin against oxidative damage to rat liver and heart tissue slices measured by oxidized heme proteins. *Journal of Nutritional Biochemistry*.

[56] Andrés D, Cascales M (2002). Novel mechanism of Vitamin E protection against cyclosporine A cytotoxicity in cultured rat hepatocytes. *Biochemical Pharmacology*.

[57] Lii CK, Ko YJ, Chiang MT, Sung WC, Chen HW (1998). Effect of dietary vitamin E on antioxidant status and antioxidant enzyme activities in Sprague-Dawley rats. *Nutrition and Cancer*.

[58] Nakamura YK, Omaye ST (2009). Vitamin E-modulated gene expression associated with ROS generation. *Journal of Functional Foods*.

[59] Pazdro R, Burgess JR (2010). The role of vitamin E and oxidative stress in diabetes complications. *Mechanisms of Ageing and Development*.

[60] Mazlan M, Sue Mian T, Mat Top G, Wan Ngah WZ (2006). Comparative effects of *α*-tocopherol and *γ*-tocotrienol against hydrogen peroxide induced apoptosis on primary-cultured astrocytes. *Journal of the Neurological Sciences*.

[61] Fatokun AA, Stone TW, Smith RA (2008). Responses of differentiated MC3T3-E1 osteoblast-like cells to reactive oxygen species. *European Journal of Pharmacology*.

[62] Varadharaj S, Watkins T, Cardounel AJ (2005). Vitamin C-induced loss of redox-dependent viability in lung microvascular endothelial cells. *Antioxidants and Redox Signaling*.

[63] Zhang P, Omaye ST (2000). *β*-Carotene and protein oxidation: effects of ascorbic acid and *α*-tocopherol. *Toxicology*.

[64] Kontush A (2001). Amyloid-*β*: an antioxidant that becomes a pro-oxidant and critically contributes to Alzheimer’s disease. *Free Radical Biology and Medicine*.

[65] Gimeno A, Zaragozá R, Vivó-Sesé I, Viña JR, Miralles VJ (2004). Retinol, at concentrations greater than the physiological limit, induces oxidative stress and apoptosis in human dermal fibroblasts. *Experimental Dermatology*.

[66] de Oliveira MR, de Bittencourt Pasquali MA, Silvestrin RB, Mello e Souza T, Moreira JCF (2007). Vitamin A supplementation induces a prooxidative state in the striatum and impairs locomotory and exploratory activity of adult rats. *Brain Research*.

[67] Maiorino M, Zamburlini A, Roveri A, Ursini F (1993). Prooxidant role of vitamin E in copper induced lipid peroxidation. *The FEBS Letters*.

[68] Huang X, Atwood CS, Hartshorn MA (1999). The A*β* peptide of Alzheimer’s disease directly produces hydrogen peroxide through metal ion reduction. *Biochemistry*.

[69] Linares GR, Xing W, Govoni KE, Chen ST, Mohan S (2009). Glutaredoxin 5 regulates osteoblast apoptosis by protecting against oxidative stress. *Bone*.

[70] Xu ZR, Hu L, Cheng LF, Qian Y, Yang YM (2010). Dihydrotestosterone protects human vascular endothelial cells from H_2_O_2_-induced apoptosis through inhibition of caspase-3, caspase-9 and p38 MAPK. *European Journal of Pharmacology*.

[71] Green DR, Reed JC (1998). Mitochondria and apoptosis. *Science*.

[72] Park SY, Cho SJ, Kwon HC, Lee KR, Rhee DK, Pyo S (2005). Caspase-independent cell death by allicin in human epithelial carcinoma cells: involvement of PKA. *Cancer Letters*.

[73] Nose K, Ohba M, Shibanuma M, Kuroki T, Pasquier C, Olivier RY, Auclair C, Packer L (1994). Involvement of hydrogen peroxide in the actions of TGFb1. *Oxidative Stress, Cell Activation and Viral Infection*.

[74] Byun CH, Koh JM, Kim DK, Park SI, Lee KU, Kim GS (2005). *α*-lipoic acid inhibits TNF-*α*-induced apoptosis in human bone marrow stromal cells. *Journal of Bone and Mineral Research*.

[75] Bai XC, Lu D, Bai J (2004). Oxidative stress inhibits osteoblastic differentiation of bone cells by ERK and NF-*κ*B. *Biochemical and Biophysical Research Communications*.

[76] Lee NK, Choi YG, Baik JY (2005). A crucial role for reactive oxygen species in RANKL-induced osteoclast differentiation. *Blood*.

[77] de Leve LD, Kaplowitz N (1991). Glutathione metabolism and its role in hepatotoxicity. *Pharmacology and Therapeutics*.

[78] Karsan A, Harlan JM (1996). Modulation of endothelial cell apoptosis: mechanisms and pathophysiological roles. *Journal of Atherosclerosis and Thrombosis*.

[79] Farré AL, Casado S (2001). Heart failure, redox alterations, and endothelial dysfunction. *Hypertension*.

[80] Then SM, Mazlan M, Top GM, Wan Ngah WZ (2009). Is vitamin e toxic to neuron cells?. *Cellular and Molecular Neurobiology*.

[81] Then SM, Wan Ngah WZ, Top GM, Mazlan M (2010). Comparison of the effects of *α*-tocopherol and *γ*-tocotrienol against oxidative stress in two different neuronal cultures. *Sains Malaysiana*.

[82] Shibata A, Nakagawa K, Sookwong P, Tsuduki T, Oikawa S, Mlyazawa T (2009). *δ*-tocotrienol suppresses VEGF induced angiogenesis whereas *α*-tocopherol does not. *Journal of Agricultural and Food Chemistry*.

[83] Birringer M, Pfluger P, Kluth D, Landes N, Brigelius-Flohé R (2002). Identities and differences in the metabolism of tocotrienols and tocopherols in HepG2 cells. *Journal of Nutrition*.

[84] Birringer M, Lington D, Vertuani S (2010). Proapoptotic effects of long-chain vitamin E metabolites in HepG2 cells are mediated by oxidative stress. *Free Radical Biology and Medicine*.

[85] Sen CK, Khanna S, Roy S, Packer L (2000). Molecular basis of vitamin E action. Tocotrienol potently inhibits glutamate-induced pp(60c-Src) kinase activation and death of HT4 neuronal cells. *The Journal of Biological Chemistry*.

[86] Khanna S, Roy S, Ryu H (2003). Molecular basis of vitamin E action: tocotrienol modulates 12-lipoxygenase, a key mediator of glutamate-induced neurodegeneration. *The Journal of Biological Chemistry*.

[87] Khanna S, Roy S, Parinandi NL, Maurer M, Sen CK (2006). Characterization of the potent neuroprotective properties of the natural vitamin E *α*-tocotrienol. *Journal of Neurochemistry*.

